# Track-based detector alignment for PET scanners

**DOI:** 10.1186/2197-7364-1-S1-A16

**Published:** 2014-07-29

**Authors:** Dennis Klöpping, David Schug, Volkmar Schulz

**Affiliations:** Department of Physics of Molecular Imaging Systems, Institute for Experimental Molecular Imaging, RWTH Aachen University, Aachen, Germany; Philips Research Laboratories, Molecular Imaging Systems, Aachen, Germany

For sub-millimeter PET the precise spatial location of each line of response (LOR) is crucial. Physical misalignments of detector modules, if not properly accounted for, can significantly degrade the spatial resolution. In this work, we investigate the application of track-based detector alignment methods for PET detectors.

We use the Hyperion-II^D^ PET/MR insert, which is composed of 10 Singles Processing Units (SPUs). Each SPU holds 2x3 stacks, each of which consists of an array of 30x30 scintillation crystals, a light guide, and an array of digital silicon photomultipliers. In principle, each crystal can be aligned separately with six degrees of freedom (3 for translations, 3 for rotations) which results in 900x6x10x6 total alignment parameters for the entire scanner. In contrast to other alignment methods proposed for PET, which suffer from potentially worse performance and efficiency, track-based alignment in general is based on the minimization of residuals by exploiting the knowledge of source positions (known with high accuracy). To demonstrate the applicability of such a method, we used the Millipede-II software package which was initially developed and is frequently used for track-based alignment in particle physics. To test the algorithm, a simple PET-Monte-Carlo simulation framework was set up: we simulated a PET system with two SPUs (six stacks each) opposite each other and applied geometric shifts (0.6 mm FWHM) to each stack on each SPU. Simulated detector data was used as input for the Millipede-II alignment software to recover the applied misalignments, which was accurately possible. An image reconstruction (ML-EM/OS-EM) using a correctly aligned and an uncorrected geometry showed significant image degradation in case of misalignments. Further investigations will include the whole detector ring with ten SPUs and rotations of each stack, as well as image reconstructions. Details about this study and experimental verification will be presented at the conference.

